# Gap junctions modulate glioma invasion by direct transfer of microRNA

**DOI:** 10.18632/oncotarget.3904

**Published:** 2015-05-04

**Authors:** Xiaoting Hong, Wun Chey Sin, Andrew L. Harris, Christian C. Naus

**Affiliations:** ^1^ Department of Cellular and Physiological Sciences, The Life Sciences Institute, University of British Columbia, Vancouver, British Columbia, V6T1Z3, Canada; ^2^ Department of Pharmacology & Physiology, New Jersey Medical School, Rutgers University, Newark, New Jersey, 07103, USA

**Keywords:** glioma, invasion, gap junction, microRNA, astrocytes

## Abstract

The invasiveness of high-grade glioma is the primary reason for poor survival following treatment. Interaction between glioma cells and surrounding astrocytes are crucial to invasion. We investigated the role of gap junction mediated miRNA transfer in this context. By manipulating gap junctions with a gap junction inhibitor, siRNAs, and a dominant negative connexin mutant, we showed that functional glioma-glioma gap junctions suppress glioma invasion while glioma-astrocyte and astrocyte-astrocyte gap junctions promote it in an *in vitro* transwell invasion assay. After demonstrating that glioma-astrocyte gap junctions are permeable to microRNA, we compared the microRNA profiles of astrocytes before and after co-culture with glioma cells, identifying specific microRNAs as candidates for transfer through gap junctions from glioma cells to astrocytes. Further analysis showed that transfer of miR-5096 from glioma cells to astrocytes is through gap junctions; this transfer is responsible, in part, for the pro-invasive effect. Our results establish a role for glioma-astrocyte gap junction mediated microRNA signaling in modulation of glioma invasive behavior, and that gap junction coupling among astrocytes magnifies the pro-invasive signaling. Our findings reveal the potential for therapeutic interventions based on abolishing alteration of stromal cells by tumor cells via manipulation of microRNA and gap junction channel activity.

## INTRODUCTION

Glioma is the most common and fatal brain cancer in adults [1]. The highly invasive nature of glioma is the primary reason for poor survival following therapeutic intervention [2]. A common pathological feature of glioma is the infiltration of reactive astrocytes [3], which are major components of the invasive niche at the interface of glioma cells and the brain parenchyma, where heterocellular signaling events occur that affect glioma progression. Astrocytes form gap junctions with glioma cells [4]; connexin43 (Cx43) is the major connexin expressed in both astrocytes and glioma cells. We have shown that Cx43 is specifically upregulated in the reactive astrocytes surrounding glioma [5], suggesting that the gap junctions between glioma cells and astrocytes at the tumor margins are involved in glioma invasion. Indeed, it has been reported that these gap junctions promote glioma invasion of the brain parenchyma [6]. How this occurs or what signals are involved is unknown. Identifying the mechanism and transferred signals would facilitate the development of novel therapies to control glioma invasion.

Gap junctions are composed of intercellular channels that uniquely allow direct movement of small signaling molecules, such as calcium ion, cyclic AMP, and phosphoinositides, between neighboring cells [7]. Since gap junctions form by the docking of hemichannels from adjacent cells, three types of gap junctions can form during cancer progression, defined by the apposed cell types: tumor-tumor, tumor-stroma, and stroma-stroma. Gap junctions between tumor cells have been studied extensively during the past 50 years, and have mostly been demonstrated to act as tumor suppressors, due to their positive effect on growth control [8, 9]. However, the role of gap junctions between tumor cells and stromal cells is less well characterized. Intriguingly, recent data suggest that they play an opposite role in cancer progression: gap junctions between cancer cells and stromal cells seems to be critical for cancer cells to invade [10, 11].

MicroRNAs (miRNAs) are small non-coding RNAs that regulate gene expression at the post-transcriptional level. The role of miRNA in cancer biology, including glioma, has been widely explored [12, 13]. Recent studies reveal that miRNAs may also function as intercellular signals in cell to cell communication, which is mediated by exosomes [14–16] or gap junctions [17–22]. Exosome mediated intercellular microRNA transfer can occur through body fluid systems, but the efficiency and specificity is unclear. In contrast, transfer of miRNA through gap junctions can avoid dilution or degradation in extracellular space, and is considered to exert more direct and targeted effects in the recipient cells [18, 23]. So far, the direct transfer of miRNA through gap junctions between adjacent cells has been demonstrated between cancer cells and stromal cells, including bone marrow stromal cells [20], mesenchymal stem cells [24] and macrophages [17]. More specifically, the exchange of miRNAs through gap junctions has been reported between glioma cells [25] and from mesenchymal stem cells to glioma cells [24]. Together, these findings raise the possibility that gap junctions modulate glioma cell invasion via movement of miRNAs between glioma cells and astrocytes.

In the present study, we systematically examined the roles of three different kinds of gap junctions in glioma invasion (glioma-glioma, glioma-astrocyte, and astrocyte-astrocyte). We demonstrate that glioma-glioma gap junctions suppress glioma invasion, while glioma-astrocyte and astrocyte-astrocyte gap junctions promote glioma invasion. Furthermore, we show that the pro-invasive effect of glioma-astrocyte and astrocyte-astrocyte gap junctions is mediated, at least in part, by the transfer of miR-5096 from glioma to astrocyte and that the effect may be amplified by signal spread among astrocytes. Our results highlight the complexity of the role of gap junctions in a tumor microenvironment and reveal for the first time that glioma cells modify stromal cells through transfer of miRNA.

## RESULTS

### Glioma-glioma gap junctions decrease glioma invasion

**Figure 1:**
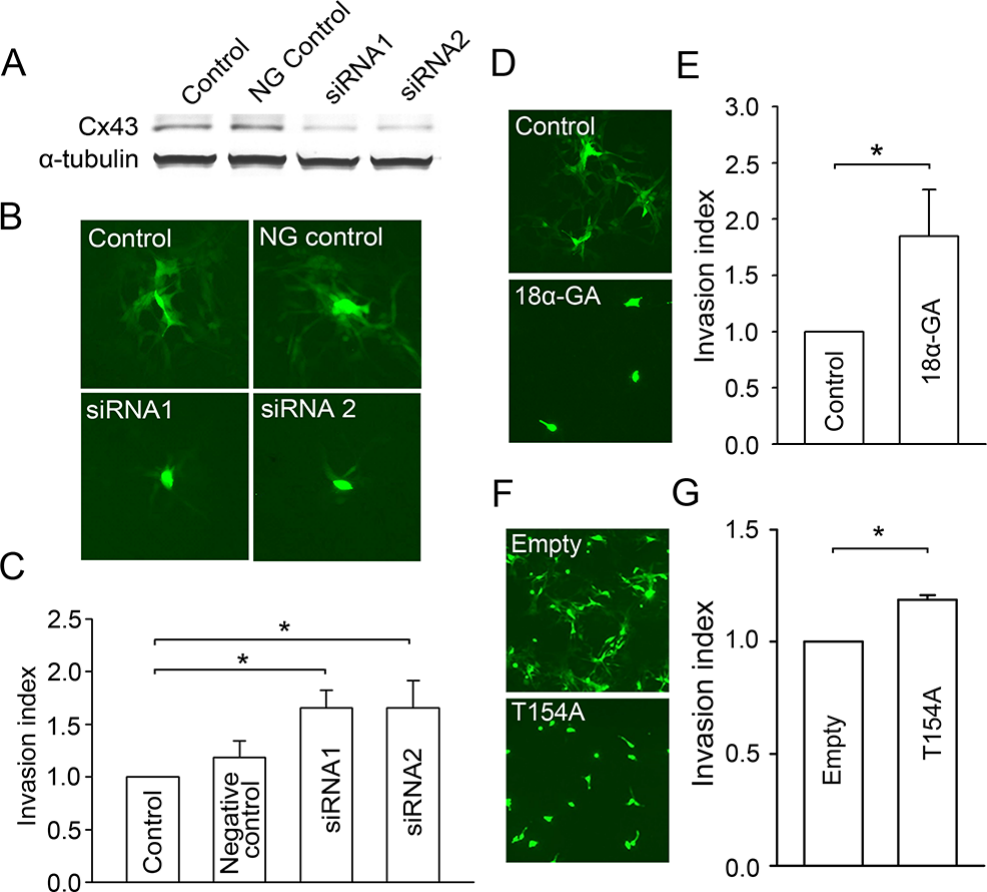
Effects of glioma-glioma gap junctions on glioma invasion **A.** Western blot showing siRNA mediated knockdown of Cx43 expression in U87MG cells. NG control: negative control siRNA. **B.** siRNA mediated knockdown of Cx43 expression in U87MG cells decreases gap junction dye coupling. Scale bar, 10 *μ*m. **C.** Effect of siRNA mediated knockdown of Cx43 in U87MG cells on glioma invasion; Cx43 siRNA increases invasion. Mean ± SEM, *n* = 4, *P < 0.05. **D.** 18*α*-GA (50 *μ*M; 1 h) effectively blocked gap junction dye coupling between glioma cells. Scale bar, 10 *μ*m. **E.** Inhibition of glioma-glioma gap junctions by 18*α*-GA increased glioma invasion. Mean ± SEM, *n* = 5, *P < 0.05. **F.** Expression of dominant-negative mutant Cx43-T154A in U87MG cells decreased gap junction dye coupling. Scale bar, 10 *μ*m. **G.** Glioma invasion is increased by expression of the dominant negative Cx43-T154A in U87MG cells. Mean ± SEM, *n* = 4, *P < 0.05.

U87MG human glioma cells express high levels of Cx43 and are widely used to investigate gap junction-dependent carcinogenesis [26, 27]. To investigate the effect of glioma-glioma gap junctions on glioma invasion, a monoculture of U87MG human glioma cells was established in a BD BioCoat matrigel transwell system. We first knocked down Cx43 expression in U87MG by using two different siRNAs. Western blotting confirmed that the knockdown of Cx43 was more than 70% with either siRNA (Figure 1A), and gap junction channel function between U87MG cells was eliminated as assessed by dye coupling (Figure 1B and Supplementary Figure S1A). More important, knockdown of Cx43 significantly increased glioma invasion (Figure 1C). Next, we used two approaches to determine whether this increase in glioma invasion is due to the loss of Cx43 protein *per se*, or more specifically, to the loss of gap junction channel function. The gap junction inhibitor 18*α*-GA [28] effectively blocked dye coupling between glioma cells (Figure 1D and Supplementary Figure S1B), and significantly increased glioma invasion (Figure 1E), suggesting that it is the lack of gap junction function rather than lack of expression of the protein that enhances invasion. To further confirm that the effect is due to the inhibition of Cx43 channel activity, we expressed a dominant negative point mutant of Cx43, Cx43-T154A [29], in the U87MG cells, which also blocked gap junction channel function (Figure 1F and Supplementary Figure S1C). Consistent with the above results, the mutant significantly increased glioma invasion (Figure 1G), confirming that decreased connexin expression itself is not responsible for the pro-invasive effect, and that the blocked junctional channel function is the key factor. Taken together, our results indicate that blocking functional glioma-glioma Cx43 gap junctions promotes glioma invasion, and conversely that functional glioma-glioma gap junctions have a suppressive effect on invasion.

### Glioma-astrocyte and astrocyte-astrocyte gap junctions promote glioma invasion

**Figure 2:**
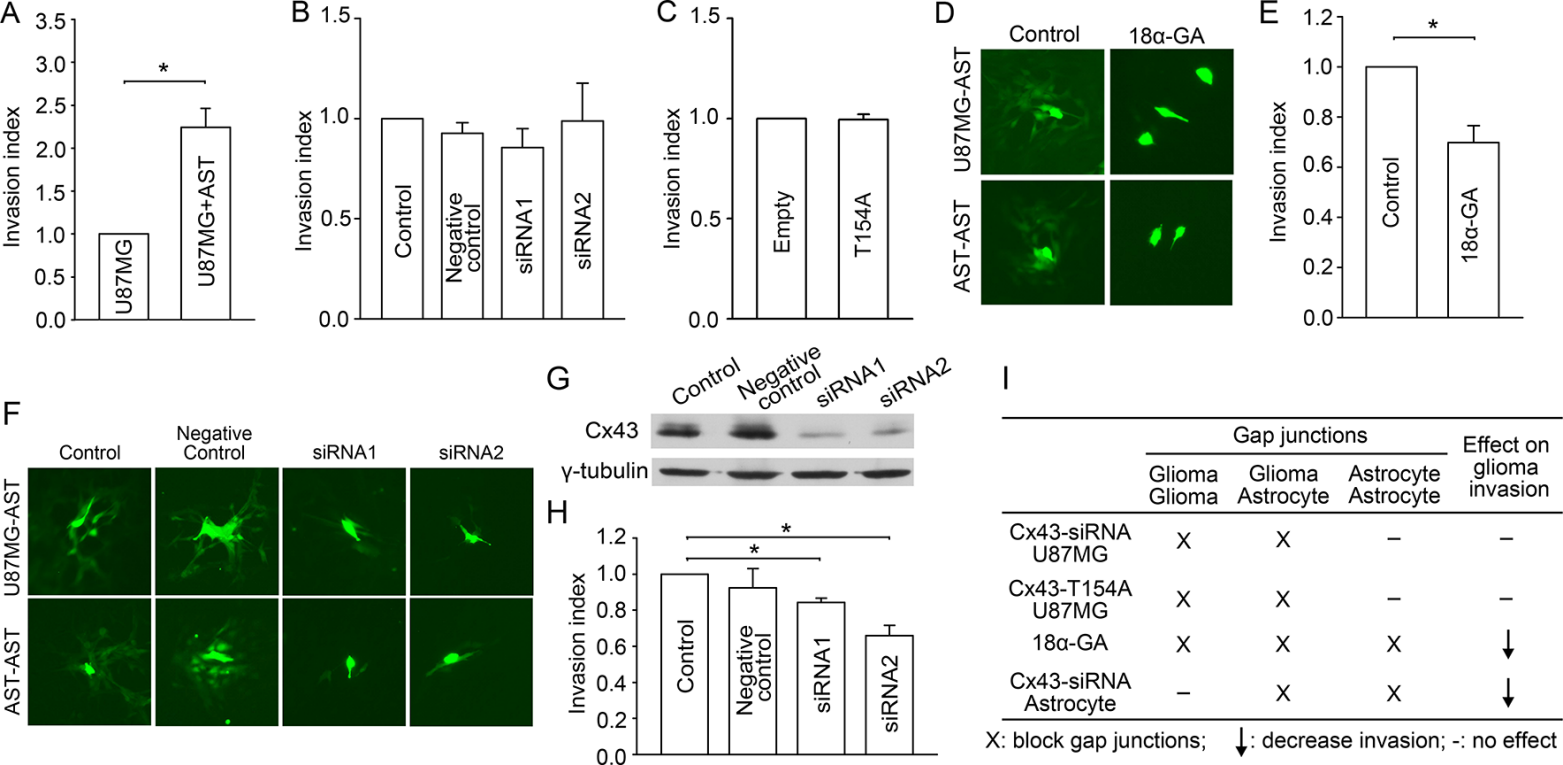
Effects of glioma-astrocyte and astrocyte-astrocyte gap junctions on glioma invasion **A.** Co-culture with astrocytes (AST) promotes U87MG cell invasion. Mean ± SEM, *n* = 5, *P < 0.05. **B.** Knockdown of Cx43 in U87MG cells by siRNAs (which inhibit glioma-glioma and glioma-astrocyte communication) did not affect glioma invasion in astrocyte co-culture. Mean ± SEM, *n* = 4, *P < 0.05. **C.** Expression of Cx43-T154A in U87MG cells did not affect glioma invasion in astrocyte co-culture. Mean ± SEM, *n* = 4, *P < 0.05. **D.** 18*α*-GA blocks glioma-astrocyte (donor: U87MG, receiving cells: astrocytes) and astrocyte-astrocyte gap junctions. Scale bar, 10 *μ*m. **E.** Block of all three types of gap junctions by 18*α*-GA decreases glioma invasion. **F.** siRNA mediated knockdown of Cx43 in astrocytes decreases glioma-astrocyte (donor: U87MG, receiving cells: astrocytes) and astrocyte-astrocyte gap junction coupling. Scale bar, 10 *μ*m. **G.** Western blot showing siRNA mediated knockdown of Cx43 expression in astrocytes. **H.** siRNA mediated knockdown of Cx43 in astrocytes decreases glioma invasion in glioma-astrocyte co-culture. Mean ± SEM, *n* = 3, *P < 0.05. **I.** Summary of different treatments to manipulate gap junction function and the final effect on glioma invasion.

Having established the inhibitory role of glioma-glioma gap junctions in invasion, we next investigated the contribution of glioma-astrocyte and astrocyte-astrocyte gap junctions to glioma invasion. To mimic the *in vivo* glioma microenvironment in which glioma cells are surrounded by astrocytes, normal human astrocytes were co-cultured with the glioma cells in the matrigel transwell, and the invasive behavior of the glioma cells was assessed. Compared to glioma monoculture, the invasive index of glioma cells is significantly increased when co-cultured with astrocytes (Figure 2A). This pro-invasive effect could be mediated by a variety of mechanisms, including glioma-astrocyte gap junctional contacts.

To explore the latter, we downregulated gap junction function in U87MG cells by siRNA-Cx43 or Cx43-T154A in this co-culture system. In addition to glioma-glioma gap junctions, glioma-astrocyte gap junctions are also blocked by these methods, but astrocyte-astrocyte gap junctions are unaffected. In contrast to the effects in glioma monoculture, the siRNAs and T154A expression had no effect on glioma invasion in the co-culture system (Figure 2B and Figure 2C). Since our previous results demonstrated that inhibition of glioma-glioma gap junctions promotes glioma invasion (Figure 1), the combined null effect on glioma invasion when, in addition, glioma-astrocyte gap junctions are blocked indicates that inhibiting glioma-astrocyte communication counteracted the pro-invasive effect of blocking glioma-glioma gap junctions. Therefore, we infer that glioma-astrocyte gap junctions promote glioma invasion.

To investigate the effect of astrocyte-astrocyte coupling in glioma invasion, we applied 18*α*-GA, which blocked all three types of gap junction coupling in the co-cultures (Figure 1D, Figure 2D, Supplementary Figure S1B, and Supplementary Figure S1D). In contrast to our finding that blocking glioma-glioma and glioma-astrocyte gap junctions has no effect on invasion (Figure 2B and Figure 2C), blocking astrocyte-astrocyte gap junctions in addition decreases glioma invasion (Figure 2E). This result indicates that astrocyte-astrocyte gap junctions (the junctions not blocked by siRNA transfection or Cx43-T154A expression in the glioma cells in Figure 2B and Figure 2C) also promote glioma invasion. That is, the combined pro-invasive effects of glioma-astrocyte and astrocyte-astrocyte gap junctions overcomes the anti-invasive effect of glioma-glioma gap junctions.

To explore this scenario further, we used siRNAs to specifically knock down Cx43 in astrocytes (rather than in glioma cells, as above), to block glioma-astrocyte and astrocyte-astrocyte gap junctions (Figure 2F and Supplementary Figure S2E), but leave glioma-glioma gap junctions unaltered. Western blotting confirmed more than 70% knockdown of Cx43 with two different siRNAs in the astrocytes (Figure 2G). Reduction of astrocytic Cx43 significantly decreased glioma invasion (Figure 2H), consistent with pro-invasive effects of glioma-astrocyte and astrocyte-astrocyte gap junctions.

Taken together, our results suggest that glioma-glioma gap junctions have anti-invasive effects while glioma-astrocyte and astrocyte-astrocyte gap junctions promote glioma invasion, as summarized in Figure 2I. We speculate that the pro-invasive effects may arise from transfer of (pro-invasive) signaling molecules from glioma cells to adjacent astrocytes via gap junctions, followed by spread of these signals (and/or their pro-invasive downstream effectors) among astrocytes through astrocyte-astrocyte gap junctions.

### Gap junctions mediate miRNA transfer between glioma cells and astrocytes

**Figure 3:**
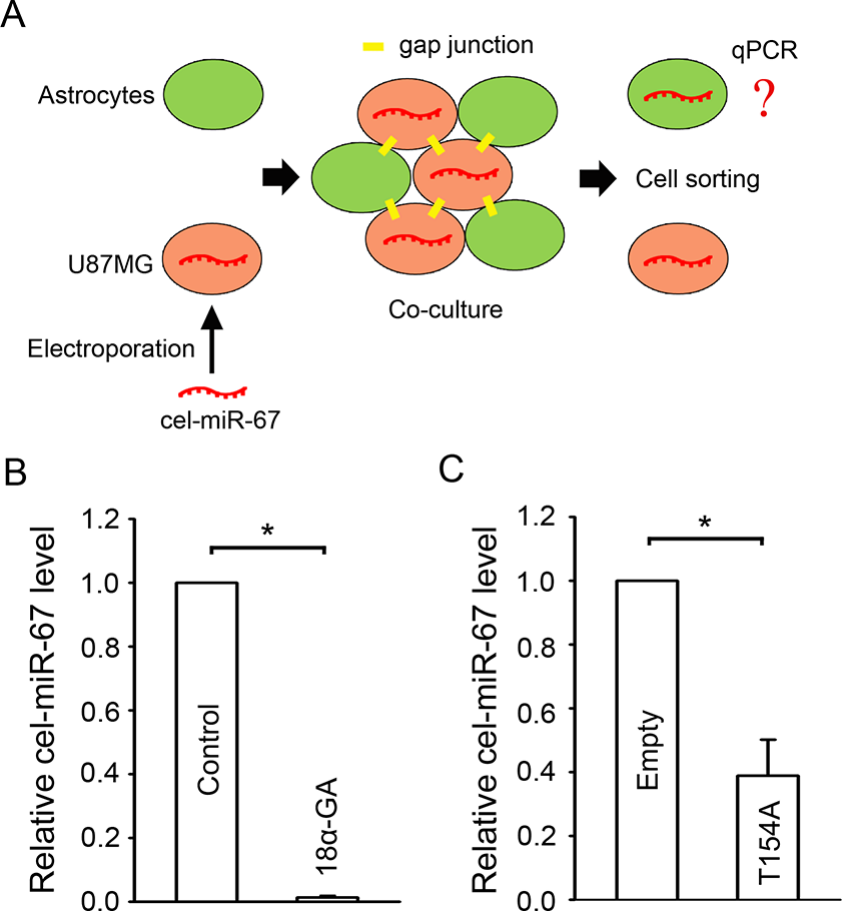
Gap junction mediated miRNA transfer between glioma cells and astrocytes **A.** Schematic diagram illustrating the procedure for confirming cel-miR-67 transfer between glioma cells and astrocytes. **B.** Normalized cel-miR-67 level in astrocytes after co-culture with U87MG cells loaded with cel-miR-67. 18*α*-GA blocks the transfer of cel-miR-67 to astrocytes. Mean ± SEM, *n* = 3, *P < 0.05. **C.** Normalized cel-miR-67 level in U87MG cells after co-culture with astrocytes loaded with cel-miR-67. The Cx43-T154A mutant greatly reduces transfer of cel-miR-67 to astrocytes. Mean ± SEM, *n* = 3, *P < 0.05.

miRNAs have been shown to pass through gap junctions between glioma cells [25] and between mesenchymal stromal stem cells and glioma cells [24]. To determine whether miRNA transfer occurs through glioma-astrocyte gap junctions, cel-miR-67, a miRNA from *C. elegans* that is not found in human cells, was employed as a “tracer”. U87MG cells were pre-loaded with cel-miR-67 by electroporation and co-cultured with astrocytes in a ratio of 1:1. The two types of cells were labeled with different Vybrant™ cell-labeling dyes, co-cultured for 24 h, and then separated by flow cytometry (Figure 3A). We detected a significant level of cel-miR-67 in astrocytes after co-culture, which was blocked by the gap junction inhibitor 18*α*-GA (Figure 3B), reducing the level of astrocyte cel-miR-67 to 1.39 ± 0.95% of control values. A similar result showing a significant level of cel-miR-67 in U87MG cells was obtained when astrocytes were loaded with cel-miR-67 and co-cultured with U87MG cells. This transfer was substantially decreased to 38.85±11.4% (Figure 3C) in U87MG Cx43-T154A receiving cells. The relatively lesser effect of the Cx43-T154A mutant in inhibiting cel-miR-67 transfer to glioma cells is consistent with its less robust effect on inhibition of gap junction coupling, relative to 18*α*-GA (Figure 1D, 1F, Supplementary Figure S2), likely due to the fact that wild type Cx43 is also being expressed in these cells. Atrocytes express other connexin isoforms, notably Cx26 and Cx30 [30, 31] that may participate in the miRNA exchange. However, Cx30 is often absent in glioma cell lines and primary glioma samples [32, 33] and U87MG cells lack the expression of Cx30 [32]. Gap junctions formed by Cx26 are reported to be not permeable to small RNAs [21]. Thus, our results confirmed the possibility of miRNA being transferred through gap junctions formed by Cx43 between glioma cells and astrocytes.

### MicroRNA profile of astrocytes is altered by glioma cells

**Figure 4:**
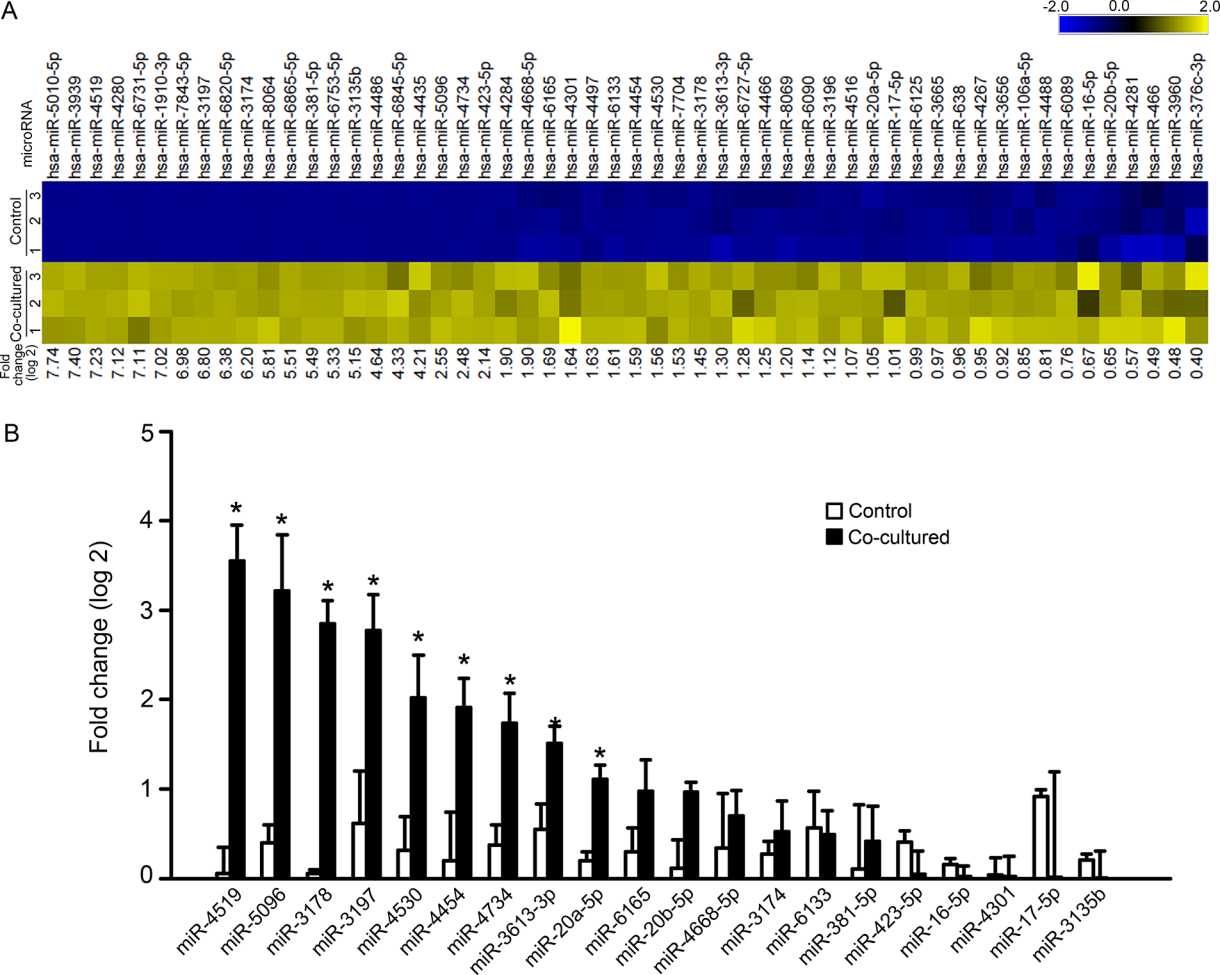
miRNA profile change in astrocytes before and after co-culture with glioma cells **A.** Heat map showing 54 miRNAs that were upregulated in astrocytes after co-culture with U87MG cells. Yellow and blue indicate relative high and low expression, respectively. **B.** qPCR analysis of the upregulated miRNAs in astrocytes before and after co-culture with glioma cells. Mean ± SEM, *n* = 3 ~ 6, *P < 0.05.

The above results raise the possibility that glioma cells deliver miRNAs to astrocytes via gap junctions, and that these miRNAs or their downstream effects subsequently spread among astrocytes via gap junctions, which amplify the pro-invasive effect. To identify potential candidate miRNAs that may be transferred to astrocytes, we first identified the miRNAs that are increased in astrocytes after co-culture with U87MG glioma cells by miRNA profiling. Total RNA from astrocytes before and after co-culture were analyzed using the *μ*Paraflo^®^ microfluidics chip, which contains all known human miRNAs (2,555 unique probes) listed in the miRBase version 20. 54 of the 2,555 miRNAs were significantly increased in astrocytes after co-culture with U87MG cells (*P* < 0.01; log2 fold change range from 7.74-0.40) (Figure 4A). We selected 25 of the 54 miRNAs for validation by real-time qPCR. The criteria for the selected miRNAs are: 1) fold change after co-culture; 2) deep sequencing data from miRBase, which indicates the annotated confidence of each miRNA [34]; 3) the number of transcript targets predicted by TargetScan, which indicates the potential biological effect of each miRNA [35]. Details for each miRNA are shown in Supplementary Table S1. We were unable to detect the expression levels of five miRNAs (miR-5010-5p, miR-3939, miR-4280, miR-4435 and miR-1910-3p) in astrocytes by qPCR. Of the remaining 20 miRNAs, 9 miRNAs showed significant increase in astrocytes co-cultured with the glioma cells (Figure 4B).

### Gap junctions mediate miR-4519 and miR-5096 transfer from U87MG to astrocytes

**Figure 5:**
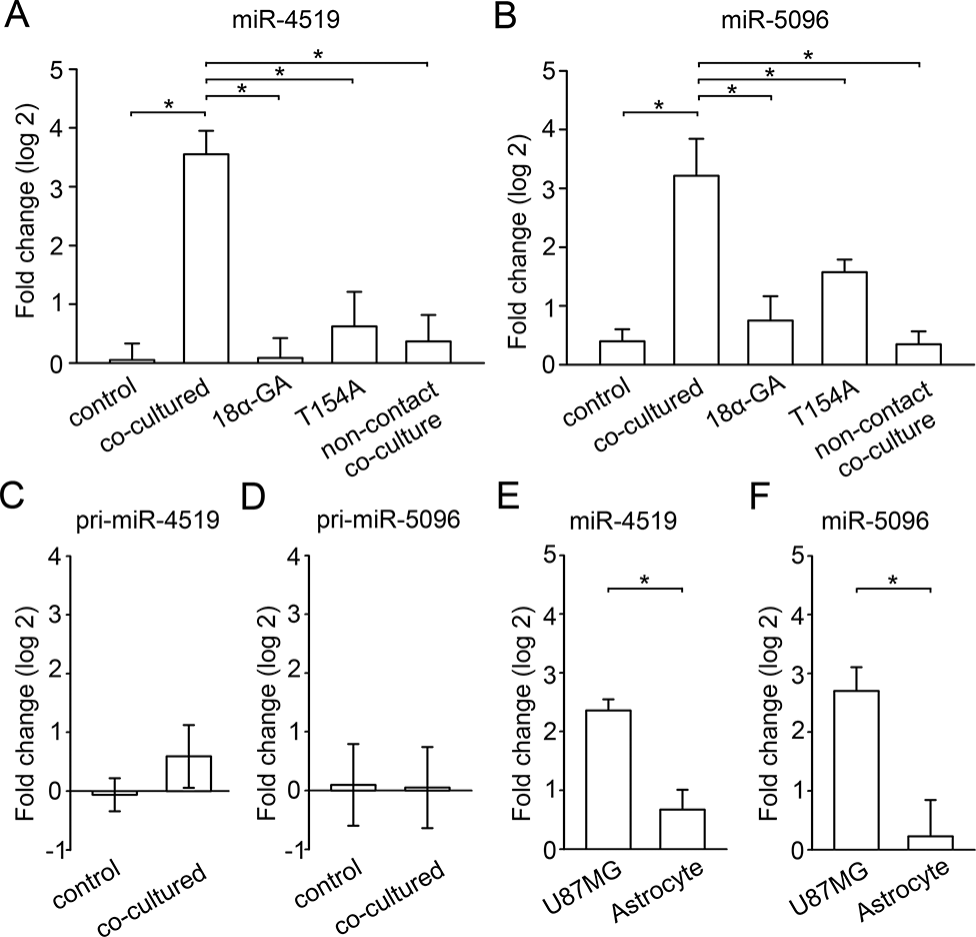
Gap junction mediated transfer of specific mature and primary miRNAs **A, B.** qPCR analysis of mature miR-4519 and miR-5096 in astrocytes before co-culture (control) or co-cultured with U87MG cells in different conditions. Mean ± SEM, *n* = 6, *P < 0.05. **C, D.** qPCR analysis of the corresponding primary miRNA levels in astrocytes before (control) and after co-culture with U87MG cells. Mean ± SEM, *n* = 3, *P < 0.05. **E, F.** qPCR analysis of levels of mature miRNAs in U87MG cells and astrocytes. Mean ± SEM, *n* = 3, *P < 0.05.

We next determined whether the increase in the levels of these 9 miRNAs in astrocytes after co-culture is gap junction dependent. qPCR was used to detect the change in miRNA levels after application of 18*α*-GA or co-culture of astrocytes with U87MG cells expressing the dominant negative channel mutant Cx43-T154A. Block of gap junction function by 18*α*-GA or Cx43-T154A completely abolished the dramatic increase of miR-4519 and miR-5096 in astrocytes that follows co-culture with glioma cells (Figure 5A and Figure 5B). In addition, non-contact co-culture was used to confirm that the effect required gap junctions. For this, glioma cells and astrocytes were cultured in the upper and bottom layers of the transwell insert, respectively, sharing the same media but without direct contact. This non-contact co-culture eliminates the physical formation of gap junction but not exosome mediated transfer. The transfer of these two miRNAs was inhibited in this condition (Figure 5A and Figure 5B, the last column to the right). Thus, our results suggest that the exchange of miR-4519 and miR-5096 depends on functional gap junctions. The other 7 miRNAs that are significantly increased after co-culture with glioma cells (Figure 4B) did not show significant change following inhibition of gap junctions.

It remains a possibility that the gap junction dependent increase in these miRNAs in astrocytes is not due to direct transfer, but is a downstream effect of junctional transfer of a different molecular signal, which induces upregulation of miR-4519 and miR-5096 within the astrocytes. If this occurred, the astrocytes would be induced to generate a large “primary miRNA” (hundreds of nucleotides to tens of kilobases long), which contains imperfectly base-paired hairpin structures that are too large to permeate gap junctions (this primary miRNA is eventually processed into the mature miRNA, which is junction permeable) [36].

To investigate this possibility, we used qPCR to detect the change in primary miR-4519 and primary miR-5096 in astrocytes before and after co-culture with glioma cells. In contrast to our findings for the mature miRNAs (Figure 5A and Figure 5B), we did not detect significant changes in the levels of primary miR-4519 and miR-5096, confirming that the increase of these two miRNAs is due to the transfer of mature miRNA from glioma cells to astrocytes, and not to stimulated expression in the astrocytes.

Gap junction mediated molecular signaling is passive, in which net signal movement results from a concentration difference. For there to be net diffusive movement of miRNA from glioma cells to astrocytes, the concentration of the miRNA must be greater in the glioma cells. We detected the expression level of these two miRNAs in U87MG and astrocytes. The levels of miR-4519 and miR-5096 are both significantly higher in monocultured glioma cells compared to astrocytes (Figure 5E and Figure 5F). Thus, our results suggest that during co-culture, glioma cells transferred the mature miR-4519 and miR-5096 to astrocytes through gap junctions.

### Elimination of miR-5096 in astrocytes decreases glioma invasion

**Figure 6:**
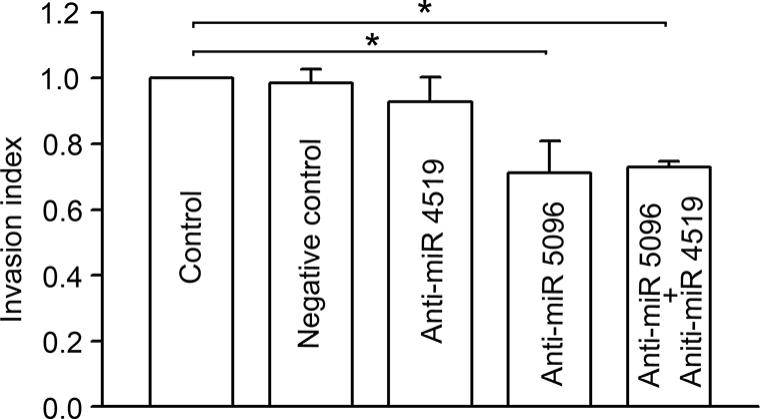
Effects of inhibition of specific miRNAs in astrocytes on glioma invasion Invasion index of U87MG cells when co-cultured with astrocytes transfected with different miRNA inhibitors (Anti-miR). Mean ± SEM, *n* = 3, *P < 0.05.

To determine whether miR-4519 or miR-5096 in astrocytes affects the invasiveness of glioma cells with which they are in contact, we transfected astrocytes with Anti-miR™ miRNA Inhibitors that specifically inhibit the function of miR-5096 or miR-4519. As shown in Figure 6, U87MG cells co-cultured with astrocytes transfected with anti-miR-5096 or co-transfected with both anti-miR-5096 and anti-miR-4519 have significantly reduced invasion compared with those co-cultured with control astrocytes or astrocytes transfected with negative control anti-miR or anti-miR-4519. These results suggest that the increase miR-5096 in astrocytes after co-culture with glioma cells is likely to contribute to the pro-invasive effect of astrocytes.

## DISCUSSION

Most studies of the role of gap junctions in cancer progression have focused on gap junctions between cells within solid tumors, with the data indicating that gap junctions between tumor cells act as tumor suppressors [8, 11]. Similar results have been reported for gliomas, in which gap junctions between glioma cells were reported to promote growth control and downregulate cell motility [37, 38]. Our results on glioma-glioma gap junctions are consistent with these findings, showing that lack of functional gap junctions between glioma cells promotes their invasiveness. Although there is some evidence that this effect in gliomas could be due to reduced cellular adhesion (because of fewer intercellular channels) [6, 39], our results show that gap junction channel activity mediates this effect. Expression of the dominant negative mutant Cx43-T154A does not interfere with the formation of gap junction plaques [29], yet it had the same effect as downregulation of the connexin via siRNA to increase the invasive behavior. Therefore, we conclude that functional glioma-glioma gap junctions led to downregulation of invasive behavior.

An increasing number of studies indicate that connexin expression and possibly gap junction channel activity have an opposite effect in the transition of cancers toward metastatic phenotypes. Connexin expression is upregulated in later stages of cancer, and in particular gap junctions between cancer cells and stromal cells promote cancer metastasis [9–11]. This phenomenon has been reported in prostate cancer [40], lung cancer [41], breast cancer [42] and melanoma [43]. As glioma cells rarely leave the central nervous system, the interaction of tumor cells with normal tissue cells plays an even more significant role in “intraparenchymal metastasis” [44], which is the hallmark of glioma. Cx43 protein has been reported to be upregulated at the leading invasion edge of the glioma cell mass [6]. Similarly, we observed increased Cx43 expression in astrocytes especially at the tumor margin where contacts between glioma cells and astrocytes occur [5]. These results indicate that Cx43-mediated communication between glioma cells and the surrounding astrocytes in the brain parenchyma is involved in glioma invasion. Indeed, functional gap junctions between glioma cells and astrocytes have been reported to promote glioma invasion [6, 39], but the mechanism by which gap junctions do so remained undetermined. Our findings support the pro-invasive role of glioma-astrocyte gap junctions, and show that astrocyte-astrocyte gap junctions enhance this process.

Signaling between glioma cells and the surrounding brain parenchyma likely involves direct mechanisms, such as gap junction intercellular communication and ligand-receptor interactions between cells, and paracrine mechanisms, such as exosomes and secreted cytokines. Empirically, our results suggest that gap junctions between glioma cells and astrocytes permit passage of molecular factors that result in enhanced invasive ability of glioma cells, and that gap junction communication between astrocytes spreads and amplifies this effect. The effect on glioma invasive capacity is likely to be due to changes in the phenotype of the astrocytes (“activated astrocytes”) [45–47], but could also involve reciprocal signaling to the glioma cells. In either case, it is the glioma-astrocyte gap junction contact that seems critical. Two key questions remain: (a) What are the molecular signal(s) transmitted through the gap junctions, which can have pro- and anti-invasive effects? (b) What are the specific changes in the astrocytes and/or glioma cells that result in changes in invasive capacity of glioma cells? Our studies focus on the former, as they seem to be causative for the latter.

Gap junction channels are permeable to a variety of cellular molecules [7]. Among the most intriguing in this context are miRNAs, particularly since miRNAs (and other single-stranded nucleotides of similar length) have been shown to permeate gap junctions formed by Cx43. The width of single-stranded RNA is 10Å, in the range of the diameter of a gap junction channel pore (10–15Å) [48]. In 2005, Valiunas et al. [21] first showed, using fluorescently labeled oligonucleotides, that single stranded RNA up to 24 nucleotides can pass through Cx43 gap junctions. Later, Katakowski et al. [25] showed that miRNA can be transferred through gap junctions between U87MG glioma cells. Recently, the transfer of miRNA through gap junctions has been demonstrated between breast cancer cells and bone marrow stromal cells [20], mesenchymal stem cells and glioma cells [24] and from macrophages to hepatocarcinoma cells [17]. In the present study, a “tracer” miRNA (cel-miR-67), not present in any mammalian cell, was used to demonstrate miRNA transfer between glioma cells and astrocytes through gap junctions. This transfer was blocked by a gap junction inhibitor and by expression of a dominant negative Cx43 channel mutant. Furthermore, by analysis of the miRNA profile change, we identified two specific miRNAs, miR-5096 and miR-4519, which were increased in astrocytes after co-culture with glioma cells, and these increases were blocked when gap junction function was inhibited using three different methods (non-contact co-culture, gap junction blocker 18*α*-GA and Cx43 dominant negative mutant T154A). The corresponding primary miRNA was not increased in the astrocytes after co-culture, showing that the synthesis of these miRNAs was not increased in astrocytes, so the miRNAs could only have come from the glioma cells. We suggest that since treatments that (a) inhibited physical formation of gap junctions, and (b) inhibited the function of gap junction plaques blocked the appearance of miR-5096 and miR-4519 in the astrocytes, the mature miRNAs were directly transferred from glioma cells to the astrocytes via gap junctions. Is this gap junction mediated transfer of miRNAs involved in modulation of glioma invasiveness? We specifically blocked these two miRNAs separately or together in astrocytes using miRNA inhibitors (anti-miR-5096 and anti-miR-4519). The glioma invasiveness was only reduced when miR-5096 was blocked in astrocytes. Thus, we conclude that glioma-to-astrocyte transfer of miR-5096 but not miR-4519 through gap junctions promotes glioma invasion.

Other mechanisms are clearly involved in regulation of glioma invasiveness. We assessed the roles of contact-mediated and non-contact-mediated effects (gap junction contacts being part of the former). A non-contact transwell in which astrocytes are cultured in the bottom well (Supplementary Figure S3) without directly contacting glioma cells was used. Contact coculture of astrocytes increased glioma invasion at 2.24 ± 0.22 fold, while non-contact transfer increased invasion 1.40 ± 0.11 fold. This indicates that both contact and non-contact mechanisms play roles in glioma invasion. The non-contact effect is possibly mediated by exosomes, which has been reported as another cell-to-cell transfer pathway for miRNA [15, 16]. However, even under conditions of restricted extracellular space, the efficiency of exosome transfer is low [18, 23]. Any effect of exosomes in the present study is likely to be in addition to the gap junction mediated effects we have shown, given the combined specificity provided by the three methods used to address gap junction function. It is unlikely that all three methods interfere with exosome secretion or trafficking. In addition, the non-contact transwell configuration would not block exosome transfer, since U87MG cells and astrocytes shared the same media. On the other hand, the transfer of the other 7 miRNA candidates that we show to be increased in astrocytes after co-culture with U87MG cells was not decreased when gap junctions were blocked. If the increase of those miRNAs in astrocytes is due to transfer from glioma cells, rather than transcriptional upregulation in the astrocytes, that transfer could be mediated by exosomes or by other secreted factors. We do not know whether any of those miRNAs exert an effect on glioma invasiveness. Further experiments are required to establish whether that is the case.

The field of miRNA biology has grown rapidly over the past ten years. Although next-generation technologies such as deep sequencing have significantly increased the rate of discovery of new miRNAs, the functions of most miRNAs are still unknown. miR-5096 is a recently discovered miRNA with no reports on its biological functions. We used TargetScan [35] (a miRNA target prediction tool), which identified 377 predicted gene targets for miR-5096. Using GENECODIS 3.0 [49], we were able to classify these genes into categories as defined by the Kyoto Encyclopedia of genes and genomes (KEGG) pathways [50]. Table S2 lists the top 13 enriched candidate regulated by miR-5096. Interestingly, by this analysis, pathways related to both “glioma” and “gap junction” are among the most ‘enriched’ by miR-5096 targets pathways. Future experiments are needed to validate the predicted protein targets of miR-5096 to understand the mechanisms by which gap junction mediated miRNA transfer regulates glioma invasion into the brain parenchyma.

In summary, our results delineate the relative contributions of different types of gap junctions, glioma-glioma, glioma-astrocyte and astrocyte-astrocyte gap junctions, in affecting glioma invasion. Furthermore, we provide evidence that glioma cells can affect stromal cells by a direct transfer of mature miR-5096 to astrocytes through gap junctions. Although further studies are required to investigate the effects of gap junction mediated miRNA transfer *in vivo*, such as using an intracranial mouse glioma model, our work suggests a key insight of the critical role of the microenvironment in tumorigenesis and provides a foundation to develop novel miRNA-based therapies for glioma control.

## MATERIALS AND METHODS

### Cell lines and cell culture

Glioma cell line U87MG was obtained from American Type Culture Collection and cultured in D-MEM supplemented with 10% fetal bovine serum; human primary astrocytes (HA1800) were obtained from ScienCell Research Laboratories (Carlsbad, CA) and were cultured according to the provider's guidelines for no more than 10 passages in astrocyte media (AM 1801). The U87MG-Cx43-T154A cell line was obtained by infection with retroviral pMSCV-puro vectors with empty plasmid or mutant Cx43 (T154A) as described previously [33]. Stable cell lines were selected in 0.75 *μ*g/ml puromycin.

### Cell invasion assay

Cell invasion assays were performed using BD BioCoat™ Matrigel™ Invasion Chamber according to the provider's guidelines with some modification. Tumor cells were labeled with cell tracker CMTPX, and added to 8 *μ*m pore transwell inserts (5 × 10^4^ cells/well in DMEM) with matrigel coating, with or without the same number of astrocytes. Medium-containing 10% FBS in the lower chamber served as chemoattractant. After 24 h, the non-invading cells were removed from the upper surface of the membrane with a cotton swab and the invading cells on the under surface of the membranes were fixed with 4% paraformaldehyde. The CMTPX labeled tumor cells from 10 random microscopic fields (160X magnification) were counted. Control experiments were carried out in the same transwell without matrigel to take account of matrigel-independent migration across the transwell membrane. The invasion index was determined as previously reported [51]. The number of cells passing through matrigel-coated membrane were divided by the number of cells passing through uncoated membrane to arrive at a number that indicates the percentage of cells (% invasion) that undergo matrigel-dependent invasion. The invasion index was subsequently calculated by dividing the % invasion of experimental cells by the % invasion of control cells.

### Dye-coupling assay

Gap junction coupling was examined as described by Goldberg et al. [52]. Cells were grown to confluence in 12-well plates. Donor cells from one well were incubated with 10 *μ*g/ml calcein-AM and 5 *μ*g/ml CM-DiI for 30 min. CM-DiI is a membrane dye that does not spread to gap junction coupled cells. Calcein-AM is converted intracellularly into the gap junction-permeable dye calcein. Unincorporated dye was removed by three consecutive washes with culture medium. The donor cells were then harvested and seeded onto the receiver cells at a 1:150 donor/receiver ratio. The cells were allowed to attach to the monolayer of receiver cells and form gap junctions for 4 h, and then examined with a fluorescence microscope. The average number of receiver cells containing calcein per donor cell was considered as a measure of the degree of gap junction coupling.

### Knockdown of Cx43 by siRNA

siRNA knockdown of Cx43 expression was performed by transient transfection with two synthetic siRNAs that target Cx43: siRNA1 (Ambion, Silencer^®^ Select siRNA, s5757, target sequence “ACUAGCUGCUGGACAUGAA”) and siRNA2 (Ambion, Silencer^®^ Select siRNA, s5759, target sequence “GAACCUACAUCAUCAGUAU”). siRNA that had no significant sequence similarity to human gene sequences (Silencer^®^ Select Negative Control No. 1 siRNA) was used as a negative control. The siRNAs were transfected into U87MG cells by Lipofectamine™ RNA max, or into astrocytes by electroporation as described below.

### Transfection of small RNAs by electroporation

Cel-miR-67 mimics (mirVana^®^ miRNA mimic, 4464066-MC22484) and miRNA inhibitors (Ambion^®^ Anti-miR™ for hsa-miR-5096(AM17000-AM22429) and hsa-miR-4519(AM17000-AM22638) were purchased from Invitrogen. Electroporation was carried out in a NEON electroporation system (Invitrogen) using the following settings: 10^7^ cells/ml, 1100 V pulse voltage, 30 ms pulse width, one pulse for astrocytes; 5 × 10^6^ cells/ml, 1300 V pulse voltage, 20 ms pulse width, one pulse for U87MG cells. Electroporated cells were transferred to antibiotic-free medium, and experiments were carried out 24 h or 48 h after transfection with miRNA (mimics and inhibitors) or siRNA, respectively.

### Western blot analysis

Cells were harvested using lysis buffer (Tris•HCl pH 7.4 20 mM, NaCl 150 mM, EDTA 1 mM, EGTA 1 mM, Triton 1%, sodium pyrophosphate 2.5 mM, Na_3_VO_4_ 1 mM, *β*-glycerophosphate 1 mM, protease inhibitors 1:1000). Monoclonal antibodies against Cx43 (Sigma, C8093, 1:5000) or *α*-tubulin (Sigma, T9026, 1:10000) were used. Immunopositive bands were visualized by Amersham ECL™ Plus Western Blotting Detection Kit (GE Healthcare), and were quantitatively analyzed using image J software.

### Co-culture of cells and cell sorting by flow cytometry

U87MG cells were labeled with Vybrant cell labeling solution DiO, and astrocytes were labeled with DiD. After labeling, cells were washed with cold PBS, and mixed together in a ratio of 1:1. After 24 h co-culture, cells were separated using a BD influx flow cytometer based on the fluorescence dye with which they were labeled. Cell sorts were carried out twice to guarantee 100% purity (Supplementary Figure S4).

### MicroRNA microarray analysis

Total RNA from astrocytes before or after co-culture with U87MG cells was isolated by mirVana miRNA isolation kit (Ambion) according to the manufacturer's protocol. RNA samples were sent to LC Sciences™ (Houston, TX, USA) for miRNA microarray analysis using the *μ* Paraflo microfluidic chips; the detailed process can be found at http://www.lcsciences.com.

### Real-time quantitative PCR for microRNA

Total RNA was isolated using the mirVana TM miRNA isolation Kit according to the manufacturer's protocol. Expression of cel-miR-67 was determined by using TaqMan miRNA assay (Invitrogen). Expression of other mature miRNAs was accessed by using miScript primer assay (Qiagen). TaqMan^®^ Pri-miRNA Assays were used to quantitate primary microRNA. The relative expression of miRNA was calculated by the comparative Ct method after normalizing to snRNA U6 (mature miRNAs) or mRNA of *β*-actin (primary miRNAs).

### Statistical analysis

The SigmaPlot version 11.0 software package was used for statistical analysis. The results are presented as mean ± standard error (SEM). Data were analyzed by one-way analysis of variance (ANOVA) or Student's *t* test. *P* < 0.05 was considered significant.

## SUPPLEMENTARY FIGURES AND TABLES


