# Supplementary material for: Gap junctions modulate glioma invasion by direct transfer of microRNA
Source: Oncotarget. 2015 May 4;6(17):15566–77. doi: 10.18632/oncotarget.3904 (PMC4558171; doi:10.18632/oncotarget.3904)
Supplement: Supplementary file 1 [file oncotarget-06-15566-s001.pdf]

SUPPLEMENTARY FIGURES AND TABLES

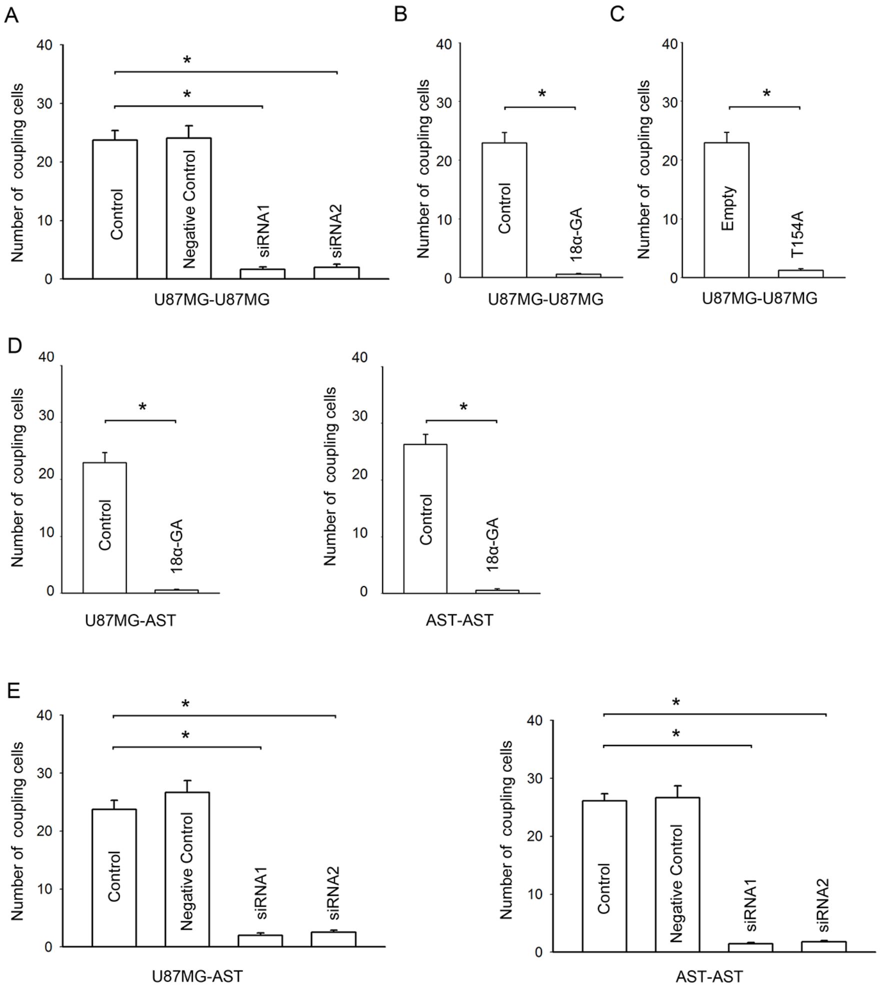

Supplementary Figure S1: Quantification data of dye coupling assay. A. siRNA mediated knockdown of Cx43 expression in U87MG cells decreases gap junction dye coupling between glioma cells. **P* < 0.01. **B.** 18*α*-GA (50 *μ*M; 1 h) effectively blocked gap junction dye coupling between glioma cells. **P* < 0.01. **C.** Expression of dominant-negative mutant Cx43-T154A in U87MG cells decreased gap junction dye coupling. **P* < 0.01. **D.** 18*α*-GA blocks glioma-astrocyte (donor cells: U87MG, receiving cells: astrocytes) and astrocyte-astrocyte gap junctions. **P* < 0.01. **E.** siRNA mediated knockdown of Cx43 in astrocytes decreases glioma-astrocyte (donor cells: U87MG, receiving cells: astrocytes) and astrocyte-astrocyte gap junction coupling. **P* < 0.01.

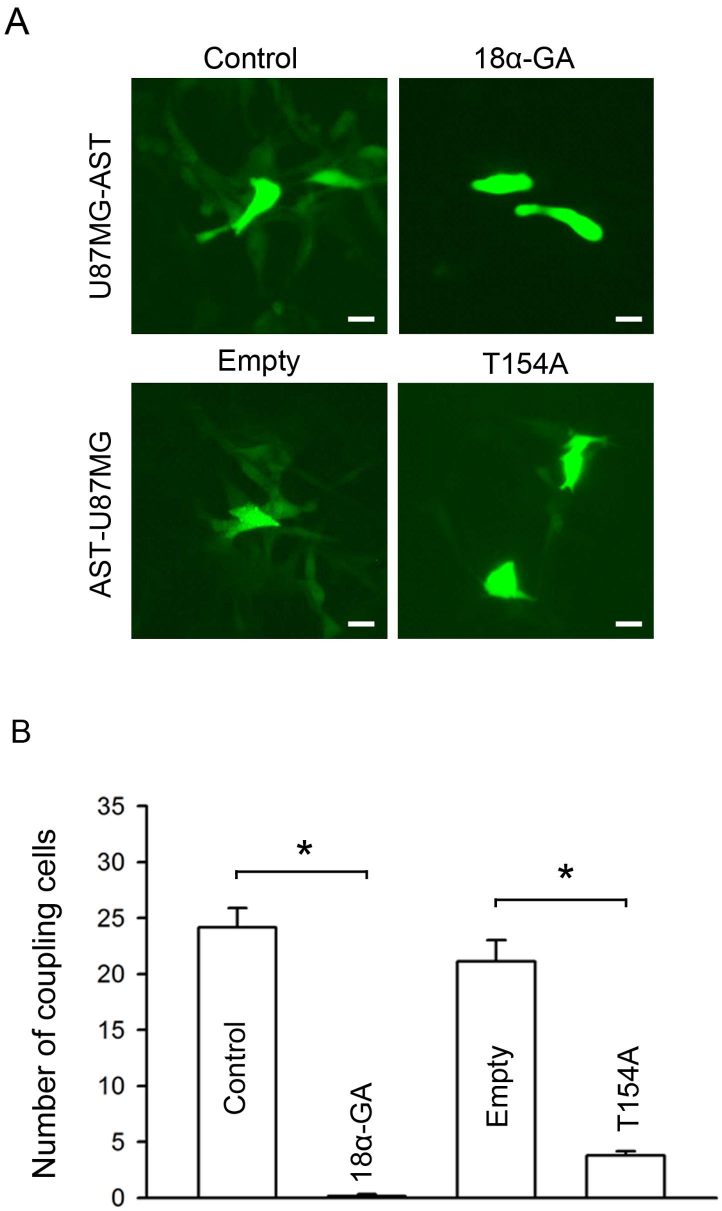

Supplementary Figure S2: Effect of 18α-GA and Cx43-T154 mutant on gap junction coupling between astrocytes and glioma cells. A. Fluorescence images showing the gap junction coupling in the presence of 18α-GA or Cx43-T154 (upper panel - donor cells: U87MG, receiving cells: astrocytes; lower panel - donor cells: astrocytes, receiving cells: U87MG) . Scale bar, 10 μm. **B.** Quantitation of glioma-astrocyte dye coupling in the presence of 18α-GA or Cx43-T154. Columns, means for 20 ~ 30 donor cells from four to six independent experiments; Bars, SEM. *$P < 0.01$.

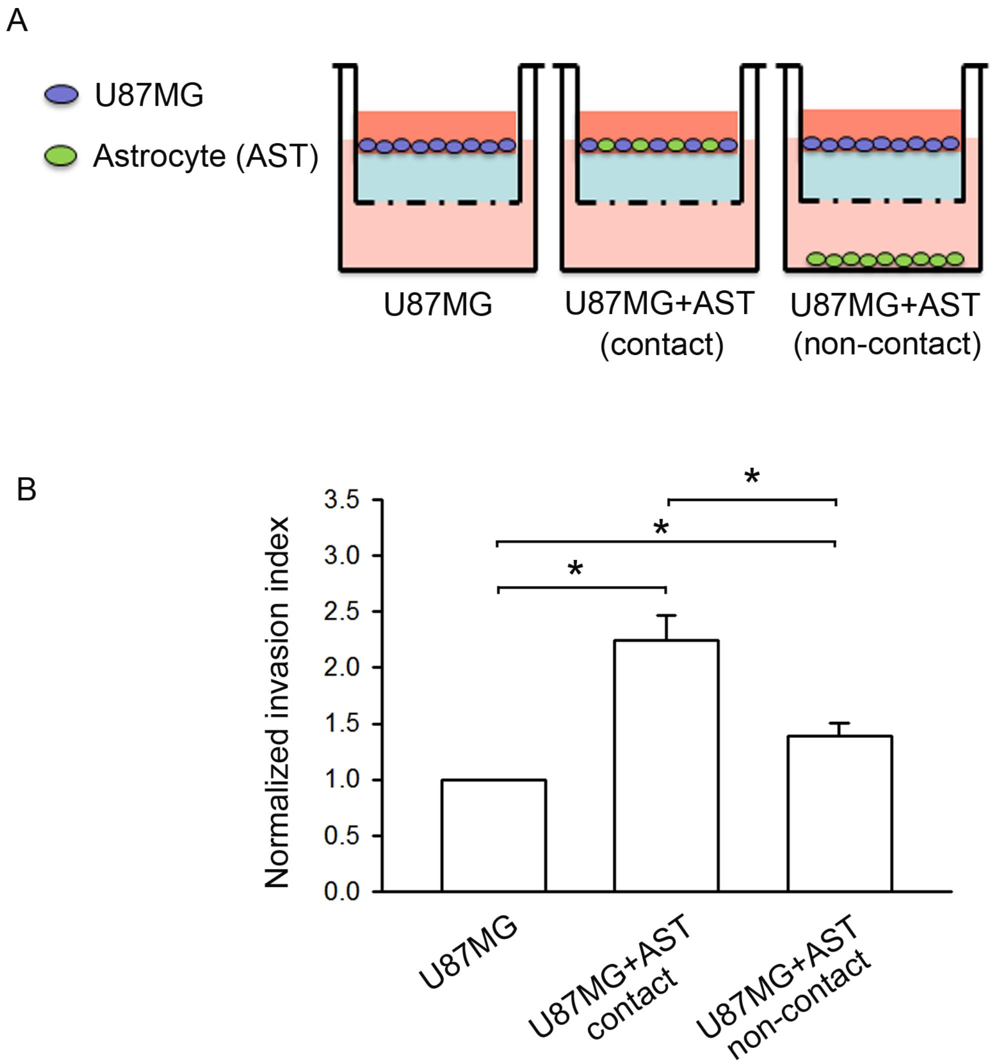

Supplementary Figure S3: Effect on glioma invasion of contact co-culture or non-contact co-culture with astrocytes. A. Schematic diagram illustrating the experimental design of contact and non-contact co-culture. **B.** Co-culture with astrocytes (AST) increases glioma invasion. Contact co-culture has much larger effects than non-contact co-culture. Mean ± SEM, $n = 3 \sim 6$, $*P < 0.05$.

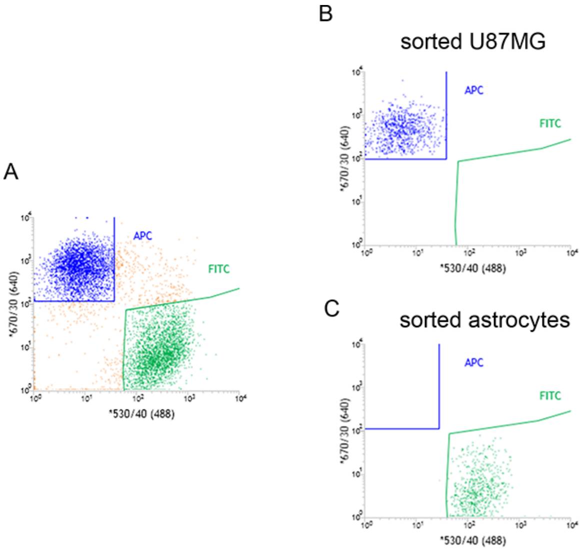

Supplementary Figure S4: Cell sorting by flow cytometry. A. U87MG cells and astrocytes were well-separated by the different fluorescent dyes with which they are labeled. U87MG cells were labeled with Vybrant DiD (APC channel) and astrocytes with Vybrant DiI (FITC channel). **B, C.** Cells were 100% pure after sorting.

Supplementary Table S1: Information of candidate miRNAs

	Fold change (log 2) ACo/ACtrl	Deep sequencing data in miRBase	Number of predicted targets in TargetScan
hsa-miR-5010-5p	7.74	710 reads, 126 reads per million, 50 experiments	0
hsa-miR-3939	7.40	19 reads, 11.4 reads per million, 8 experiments	3
hsa-miR-4519	7.23	11 reads, 23.9 reads per million, 12 experiments	322
hsa-miR-4280	7.12	11 reads, 46.2 reads per million, 6 experiments	164
hsa-miR-6731-5p	7.11	1 reads, 0.2 reads per million, 1 experiments	0
hsa-miR-1910-3p	7.02	139 reads, 72.6 reads per million, 33 experiments	301
hsa-miR-7843-5p	6.98	1 reads, 0.0977 reads per million, 1 experiments	0
hsa-miR-3197	6.80	17 reads, 184 reads per million, 21 experiments	13
hsa-miR-6820-5p	6.38	no data	0
hsa-miR-3174	6.20	45 reads, 15 reads per million, 26 experiments	113
hsa-miR-8064	5.81	1 reads, 0.748 reads per million, 1 experiments	0
hsa-miR-6865-5p	5.51	1 reads, 0.222 reads per million, 2 experiments	0
hsa-miR-381-5p	5.49	1091 reads, 486 reads per million, 44 experiments	877
hsa-miR-6753-5p	5.33	14 reads, 0.893 reads per million, 4 experiments	0
hsa-miR-3135b	5.15	280 reads, 316 reads per million, 46 experiments	322
hsa-miR-4486	4.64	99 reads, 24.1 reads per million, 13 experiments	82
hsa-miR-6845-5p	4.33	1 reads, 0.2 reads per million, 1 experiments	0
hsa-miR-4435	4.21	142 reads, 69.5 reads per million, 13 experiments	337
hsa-miR-5096	2.55	1993 reads, 778 reads per million, 64 experiments	377
hsa-miR-4734	2.48	54 reads, 83.9 reads per million, 29 experiments	18
hsa-miR-423-5p	2.14	167693 reads, 8780 reads per million, 80 experiments	180
hsa-miR-4284	1.90	1046 reads,1490 reads per million, 58 experiments	339
hsa-miR-4668-5p	1.90	34 reads, 114 reads per million, 24 experiments	552
hsa-miR-6165	1.69	21 reads, 136 reads per million, 16 experiments	0
hsa-miR-4301	1.64	37 reads, 67.8 reads per million, 23 experiments	0
hsa-miR-4497	1.63	819 reads, 3260 reads per million, 64 experiments	12
hsa-miR-6133	1.61	1161 reads, 330 reads per million, 45 experiments	0
hsa-miR-4454	1.59	3121 reads, 1570 reads per million, 69 experiments	7
hsa-miR-4530	1.56	11 reads, 116 reads per million, 13 experiments	547
hsa-miR-7704	1.53	127 reads, 5.95 reads per million, 5 experiments	0
hsa-miR-3178	1.45	512 reads, 1360 reads per million, 55 experiments	10
hsa-miR-3613-3p	1.30	34 reads, 62.4 reads per million, 24 experiments	2635
hsa-miR-6727-5p	1.28	1 reads, 0.171 reads per million, 1 experiments	0
hsa-miR-4466	1.25	132 reads, 502 reads per million, 46 experiments	6

(*Continued*)

	Fold change (log 2) ACo/ACtrl	Deep sequencing data in miRBase	Number of predicted targets in TargetScan
hsa-miR-8069	1.20	0	0
hsa-miR-6090	1.14	27 reads, 172 reads per million, 28 experiments	0
hsa-miR-3196	1.12	472 reads, 791 reads per million, 56 experiments	50
hsa-miR-4516	1.07	988 reads, 3.24e+03 reads per million, 59 experiments	423
hsa-miR-20a-5p	1.05	8073 reads, 4210 reads per million, 73 experiments	1220
hsa-miR-17-5p	1.01	16202 reads, 7380 reads per million, 78 experiments	1220
hsa-miR-6125	0.99	145 reads, 427 reads per million, 44 experiments	0
hsa-miR-3665	0.97	1832 reads, 3770 reads per million, 60 experiments	292
hsa-miR-638	0.96	51 reads, 227 reads per million, 30 experiments	30
hsa-miR-4267	0.95	3 reads, 29 reads per million, 7 experiments	442
hsa-miR-3656	0.92	1007 reads, 2600 reads per million, 58 experiments	21
hsa-miR-106a-5p	0.85	10053 reads, 4410 reads per million, 74 experiments	1220
hsa-miR-4488	0.81	1660 reads, 1590 reads per million, 59 experiments	68
hsa-miR-6089	0.76	107 reads, 350 reads per million, 45 experiments	0
hsa-miR-16-5p	0.67	36647 reads, 3930 reads per million, 74 experiments	1273
hsa-miR-20b-5p	0.65	10305 reads, 1300 reads per million, 65 experiments	1220
hsa-miR-4281	0.57	34 reads, 332 reads per million, 34 experiments	203
hsa-miR-466	0.49	47 reads, 65.9 reads per million, 17 experiments	680
hsa-miR-3960	0.48	1550 reads, 4680 reads per million, 67 experiments	34
hsa-miR-376c-3p	0.40	2784 reads, 1350 reads per million, 47 experiments	254

Note: the selected miRNAs for further validation were underlined.

Supplementary Table S2: Top 13 enriched pathways regulated by miR-5096

Pathway names	*P* value	No. of Genes	Gene names
Pathways in cancer	1.66E-05	17	SOS1; MET; BCL2L1; RUNX1T1; TPM3; PDGFA; CDKN1B; PIK3R3; LAMA4; PIK3R1; FZD4; FZD3; KRAS; APPL1; RET; XIAP; RXRA
ErbB signaling pathway	3.21E-05	9	SOS1; CAMK2D; SHC3; EREG; MAP2K4; CDKN1B; PIK3R3; PIK3R1; KRAS
Focal adhesion	8.71E-05	12	SOS; MET; SHC3; THBS1; PDGFA; PIK3R3; LAMA4; PIK3R1; ACTN2 COL5A1; XIAP; PPP1CC
Glioma	1.92E-04	7	SOS1; CAMK2D; SHC3; PDGFA; PIK3R3; PIK3R1; KRAS
Endocytosis	2.64E-04	11	RAB22A; MET; DNAJC6; NEDD4L; DNM3; RET; RAB11FIP2; ACAP2; LDLRAP1;
Chronic myeloid leukemia	3.43E-04	7	SOS1; BCL2L1; SHC3; CDKN1B; PIK3R3; PIK3R1; KRAS
Fc epsilon RI signaling pathway	4.18E-04	7	SOS1; MAP2K4; PIK3R3; PRKCE; PIK3R1; MAPK14; KRAS
Chemokine signaling pathway	5.51E-04	10	SOS1; ADCY6; SHC3; ADCY1; GNB4; PIK3R3; WASL; PIK3R1; KRAS; CXCL12
MAPK signaling pathway	5.82E-04	12	RASGRP3; CACNG2; SOS1; NLK; MAPT; MAP3K12; PDGFA; MAP2K4; TAOK1; SRF; MAPK14; KRAS
Neurotrophin signaling pathway	2.47E-03	7	SOS1; CAMK2D; SHC3; PIK3R3; PIK3R1; MAPK14; KRAS
Gap junction	2.55E-03	6	SOS1; ADCY6; ADCY1; GRM5; PDGFA; KRAS
Regulation of actin cytoskeleton	2.78E-03	9	SOS1; EZR; PDGFA; PIK3R3; WASL; PIK3R1; ACTN2; KRAS; PPP1CC
Adherens junction	4.49E-03	5	MET; NLK; WASL; CTNND1; ACTN2
